# 

**Published:** 1890-01

**Authors:** 


### TABLE OF CONTENTS.

### 1890.

PAGE

A Queen's Prescription .... .. .......... ... 12
A Hygienic Necessity.... ............... ..... 17
A Dangerous Drug... ...................... 39
A Mystery . . .......... .. ........ ...... 41
A Social Reform from the Kitchen...... ... 42
A Good Test for Coffee ................... . 43
Apoplexy ..................................... 56
A Mistake to Exercise for Strength alone ... 60
A Strange Coincidence .................. ... 62
A Doctor's "Don'ts".. ..................... 78
A Test of Death ................. ......... 83
A Sanitary Wash-House .... ...... ........ 84
A Wonderful Flower... ... .... .. ........ 91
A Change for the Better ........ .......... 97
Amusements ... .. ............ ........... 97
Are New Yorkers Losing their Eyesight.....103
A Mummy Unrolled........... ..... .......113
A Cheap Filter.... ............. ......... .. 127
A Cause of Disease.... ............. ..... ...134
Alcoholic Adulteration ......................145
Apples as Medicine ... ........................152
A Presentiment....... ............ ... ......153
Another Boy Prodigy .......................156
A Pathetic Scene ............ ... .........158
A Gratifying Acknowledgment.. ............163
A Girl's Training .......................... 196
A Wish on the Moon....... ..................216
An Athlete's Diet ........................ .......178
A Boy's Temper ........ . ............... 185
An Indian's Argument for Vegetarianism ... 188
A Tidy and Healthful Home .................223
Amazing Virulence of Germs .......... .....230
Arsenic and Ammonia .... ...................239
All Forms of Life Cellular ............ ... ..235
Anxiety......................................... 82
A Monkey in Church... ..... ... ........... 279
A Cold in the Head ............ ..............275
A Practical Philanthropist ....................265

Bacilli on a Bald Head........................ 68
Beer as a Tonic . . ..........................194
Born with a Chill ...............................260
Botch Bosses . ..... .........................124
Boston Baked Beans ...........................211
Born-Blindness Preventable................. 180

Confectioners' Colors ........ ............... 60
Concerning Food ..... .. ........ ......... ..... 96
Calisthenics ....................... ..............100
Cod Liver Oil .....................................105
Colic ... ............... .........................112
Candies Made of White Clay................. 163
Cure for Drunkenness ................... ... 164
Convulsions .. ..... ................ ......200
Chemicals for Household Use ... ............200
Co-operative Housekeeping ..................203
Cheerful Talk........................ ..... ...224
Cod Liver Oil Substitute ...... ...............231
Children's Teeth ........... .... ............234
Care of the Skin .................................232
Corpulency .... ............ ......... ...254
Catarrh .............................................259
Consolation ................ .. .. ...........264
Cancer and Smoking.......... . ... .........277
Children's Eating........ ....................274
Curious Effects of Poisons. ... ...............271

Diseases from a Book........ ... .......... 45
Digestion .......... .. . ...... ........ ... 82
Defective Sense........ ....................... 132
Dr. Mary Walker ............................ ..137
Diphtheria in Chewing Gum ............. ..236
Drugged Baking Powders................. .262

PAGE

Exercise and Cleanliness............ ....... 6
Effect of Beer Drinking ........ ............ 67
Eczema........ ........ ........ ............... 80
Eggs. . ...........................................106
Electric Currents of the Skin... .... ... ...110
Evil Effects of Catarrh ..................... ..159
Early History of the Saw ....................209
Extracting Foreign Bodies from the Stomach179
Effects of Quinine ..............................280

Farmers and Farming....................... 54
Fasting ................. ......................... 85
For Sea Sickness .... ...................... ..227

Good Rules for Dyspeptics....... .... .....107
Government Getting us Good Groceries... .150
Great Times for the Widows. . ..... ......156
Goldfish have Fun with the Turtle ..........187
Get Back in De Ribber..........................282

Home Training of Girls ...................... 11
Household.... ......... ...21-46-70-92-118-139
How and When to Drink Water. ..... ..... 42
How to Elude the Doctor ............ ...... 69
How Long to Sleep ............................ 77
How to Walk and Sit .. .... .. ....... ....108
Hygiene of Carpets ... ....... ..............116
Hygiene of Laughter..................... ..128
Hunger .......................................... .. 133
How Monkeys are Captured.................201
Home-Made Soap ............... ... ..........232
How to Keep the Baby Well....... ... .....256
Home the Place to Play........ .......... 229

Infant Damnation........ ............... ..... 44
Insomnia ........................... ............. 63
In the Sick Room. ............................ 68
Ideas of a Future Life ... .. .............. 87
Infection in Chewing Gum.... ............ ..126
Is Crime a Disease . ...... ... .............197
Infant Foods ............ .....................208
In the Period of Expectation.................251

Japanese Morals ....... ....... .........279

Kindness to Animals... ........ ........182
Keep the Mouth Closed.......................278

Literary ...23-48-72-93-120-143-167-215-192-240-263-287.
Looking Forward............ ............ 2
Letting Babies Walk too Early .............. 20
Looking Forward..... ........... ....... 26
Looking Forward More Truly.... .. .. .... 31
Laughter as a Health Promoter ..... ....... 43
Looking Forward....................... .... 73
"Lay Not Thy Hand to the Plowing Beast" 79
Love and Home............. ... ...........244

Miscellaneous..22-47-71-93-118-141-163-186-210-237-260-284.
Mummified Cats ..... ..... ............. 88
Medicinal Uses of Coffee.......... ........ 111
May . .. ..........................................120
Mesmerism . .. ............. .... .......128
Mind Reading ...................... .......157
Molly Fancher's Bazaar... ..................161
Mesmerism ..................... ..............169
Moths ............................ ......... ...181
Maiden Fair .... ...... ........................240
Mother and Offspring................... ....257

National Sisterhood of Liberal Women...... 25

| | PAGE |
|---|---|
| National Liberal Women's Suffrage Association | 38 |
| Nature's Demands | 121 |
| Nerves and Cells | 248 |
| Neatness in Dress at Home | 280 |
| Old and Young Sleeping Together | 64 |
| On the Strumous Diseases of Childhood | 222 |
| Pure Air | 67 |
| Poison for Arrow Tips | 161 |
| Private Benevolent Societies and the Census | 162 |
| Paste for Scrap Books | 165 |
| Poisoning by Tinned Fruit | 180 |
| Proper Clothing | 204 |
| Physical Culture | 205 |
| Preserving the Health | 210 |
| Physical Education in Relation to Mental Development | 225 |
| Paralysis and Apoplexy | 253 |
| Quinine Intoxication | 179 |
| Rev. M. J. Savage on Occultism | 35 |
| Removal of Moles by Electricity | 43 |
| "Remedies" | 44 |
| Recasting the Creed | 49 |
| Round Shoulders | 111 |
| Recent Floods | 129 |
| Red Noses | 281 |
| Sleep | 91 |
| Sherry Wines | 135 |
| Sulphur in Sugar | 207 |
| Salt for Moths | 213 |
| Swimming Baths for Children | 228 |
| Source of Colors | 64 |
| Sacred Trees | 282 |
| The New Year | 1 |
| Trichinosis | 9 |
| The Effects of Tight Clothing | 18 |
| The Care of Infants | 19 |
| The Laws of Health | 20 |
| The Walled Lake | 21 |
| The Feet | 15 |
| The Christian Buddha | 17 |
| The Source of Wrinkles | 45 |
| The Drinking Habit | 50 |
| To a Hopeless Dyspeptic | 51 |
| Troubled Topolobampo | 57 |
| The Office of Iron in the Blood | 63 |
| The Ear | 67 |
| The Art of Prolonging Life | 88 |
| The Fear of Death | 81 |
| The Danger of Tea Drinking | 86 |
| The Invisible World | 86 |
| The Nose | 89 |
| The Canal of Joseph | 109 |
| Trephining for Insanity | 110 |
| The Educational Value of Out-door Games | 114 |
| The Goldsmith Piano | 115 |
| The Fever Microbe | 116 |
| The Liver | 123 |
| The Cure of Consumption | 126 |

| | PAGE |
|---|---|
| The Working Girl | 130 |
| The Human Will | 131 |
| The Acids of Fruits | 135 |
| Tea-Making | 136 |
| The Skin | 137 |
| The Regeneration of the Body | 138 |
| The Alpine Rose | 144 |
| The Eyes | 147 |
| True Worth Recognized | 155 |
| The Invisible World | 154 |
| The Potato | 164 |
| The Nose | 159 |
| The Hygiene of Motherhood | 241–266 |
| Tooth Powders and Tooth Washes | 246 |
| The Requisites of Food | 255 |
| The First Chew | 252 |
| The Ear | 208 |
| The Art of Bread Making | 212 |
| Ten Good Things to know | 215 |
| The Summer Outing | 193 |
| Treatment of Sunstroke | 202 |
| To Detect Adulteration of Milk | 231 |
| The Art of Prolonging Life | 233 |
| The Death Penalty | 217 |
| The Science of Hygiene | 219 |
| The Husband's Part | 221 |
| Torpid Liver | 171 |
| The Occult Powers | 173 |
| The Proper Weight | 174 |
| The Stenographic Art | 175 |
| Tea and Coffee | 176 |
| The Tongue | 181 |
| The Art of Prolonging Life | 184 |
| The Spread of Consumption | 184 |
| The Mystery of the Magnet | 186 |
| Tell Me, My Heart | 192 |
| The Use of Water at Meals | 283 |
| The Egg a Remedy for Dysentery | 274 |
| The Fair Theosophist | 288 |
| The Teeth | 270 |
| The Corset | 273 |
| Ventilation | 16 |
| Various Uses for Ammonia | 38 |
| Vegetarianism | 115 |
| Valor and Skill in the Civil War | 129 |
| Woman | 40 |
| Would you like a New Nose ? | 61 |
| Where some Nuts come from | 65 |
| Wheat Meal vs. White Flour | 59 |
| What's in a Dream | 83 |
| Why the Sea is Green | 90 |
| Woman's Dress | 112 |
| Well Water | 131 |
| Whitewash for Ceilings | 138 |
| What Man is Made of | 155 |
| We Eat Too Much | 198 |
| Woman | 190 |
| What Exercise Does | 227 |
| What Education is Doing for Us | 206 |
| Wonderful Coal Products | 206 |
| What to Eat in Cold Weather | 276 |
| Woman's Dress | 277 |

### LITERARY.

*The Youths' Companion.* The Christmas number of this excellent publication is simply gorgeous. It is, as it deserves to be, the leading youths' paper in the field Its illustrations, too, are always equal to ihe highest state of the art, and plenty of them. Perry Mason & Co,, Boston.

*A Unique Business Calendar.* A most convenient, valuable and unique business calendar for 1890, is the Columbia Bicycle Calendar and Stand, issued by the Pope Mfg. Co., of Boston. Mass. It is in the form of a pad of 366 leaves, 5⅛x3¾ in., one for each day of the year, and one for the entire year. A good portion of each leaf is blank for memoranda.

*Lend a Hand* is one of the most tasty and ably conducted of our exchanges.—Boston : J. S. Smith & Co., 3 Hamilton street ; monthly.

*The Autobiography of Joseph Jefferson*, the Rip Van Winkle of the play house, now being published in the *Century Magazine*, bids fare to rival in interest the most popular articles of this excellent periodical.

We have received the report of the 22d annual meeting of the American Otological Society, which met at New London, Conn., July, 1889. It is very handsomely gotten up, and contains the names of enough fellow M. Ds. to stock a hundred grave yards.

We are in receipt of a copy of J. H. Kob's New Musical System, advertised in this issue. It is claimed to be a great improvement on the old.

The Open Court Publishing Company, of Chicago, announces the immediate appearance of the authorized translation of M. Th. Ribot's *Psychology of Attention* The monograph of M. Ribot, who is now Professor of Experimental and Compara- Psychology at the College de France, and editor of the *Revue Philosophique*, has been characterized by a prominent French critic as the most important production of the Frencn Philosophical press for the preseut year. Open Court Publishing Company, 169-175 La Salle Street, Cdicago, Ill.

*The Estey Family*, by Sara E. Hervey is the story of a decidedly commonplace group, who deliver homilies to each other on all occasions, and who are all most comfortably married off by the time the last chapter is reached. The story is garnished with poetical quotations from various authors together with dialogues morally irreproachable. Published by the author, Onset, Mass, Price, post-paid, $1.07. Page 276.

